# Breath of green life: Reduction in plant day and night respiration under elevated CO_2_

**DOI:** 10.1093/plphys/kiad087

**Published:** 2023-02-14

**Authors:** Alexandra J Burgess

**Affiliations:** Assistant Features Editor, Plant Physiology, American Society of Plant Biologists, USA; Agriculture and Environmental Sciences, School of Biosciences, University of Nottingham Sutton Bonington Campus, Loughborough LE12 5RD, UK

Plants constantly respire, even when photosynthesizing. Current estimates indicate that half the amount of carbon assimilated via terrestrial vegetation is subsequently lost during respiration (Dusenge et al. 2019). Net primary production (NPP), the total carbon (C) fixed by photosynthesis minus the total fixed C lost by respiration, represents the rate at which energy is stored as biomass by plants and made available to the consumers in an ecosystem.

Although NPP may seem a simple parameter to determine, in the real world, the rates of both respiration and photosynthesis are differentially affected by multiple factors including water, temperature, carbon dioxide concentration (CO_2_), and nutrients. For example, while photosynthesis is known to be stimulated under elevated (CO_2_), termed the “fertilization effect,” the corresponding impact on respiration is poorly understood. This has implications for global models of carbon balance, carbon use efficiency, and ecosystem function under climate change (Atkin et al. 2017; Tcherkez et al. 2017).

Another complication in assessing NPP relates to the fact that plant respiration occurs both in the light (*R_l_*) or the dark (*R_d_*). Whilst light partially inhibits leaf respiration, the contribution of *R_l_* versus *R_d_* to CO_2_ evolution varies greatly (Crous et al. 2017; Murchie et al. 2022). This is partly a result of day length and differential day versus night temperature in most environments, as well as physiological status and variations in C and nitrogen (N) metabolism between species (Atkin et al. 2007; Gong et al. 2017). However, the specific response of each to changes in conditions is not fully understood, and conflicting responses of *R_l_* to increasing atmospheric (CO_2_) have been found.

**Figure 1.**
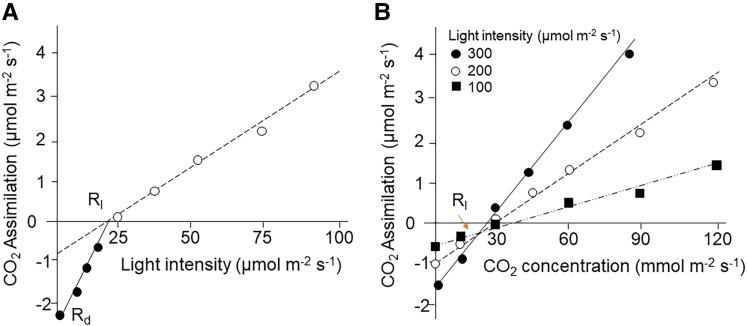
Conceptual overview for the calculation of day respiration (*R_l_*) according to two different methods. **A)** The Kok method (Kok 1949), where the open and closed symbols indicate the different points used to fit the biphasic relationship between CO_2_ assimilation and low light intensity, above and below the light compensation point, and *R_d_* indicates the dark respiration rate. **B)** The Laisk method (Laisk 1977) where the different colored symbols indicate measurements under different light intensities. Adapted from Yin et al. (2011). N.B. data are shown for illustrative purposes only and do not come from real measurements.

One potential reason for the inconsistent reports is a result of the difficulties in measuring *R_l_* and *R_d_*. The two most common methods used require manipulation of assimilation rates (*A*) under low irradiance (i.e. less than 150 µmol m^−2^ s^−1^; termed the Kok method, Fig. 1A; Kok 1949) or under low CO_2_ (called the Laisk method, Fig. 1B; Laisk 1977); the latter of which is unsuitable for determining the response of respiration to altered (CO_2_). Previous studies have shown that the Kok effect (referring to the breaking point, or rapid decrease in CO_2_ assimilation around the light compensation point) is not caused exclusively by changes in respiration (Gauthier et al. 2020). The Kok method also assumes a constant Photosystem II efficiency (Φ_2_) which often results in a lower estimate of *R_l_* relative to the Laisk method. However, the incorporation of chlorophyl fluorescence measurements, proposed by Yin et al. (2011), can account for the decline in PSII electron transport efficiency with increasing light intensity. This adapted methodology is known as the Kok-Phi or Yin method. Similarly, the Kok method also assumes a constant chloroplastic (CO_2_) (*Cc*) throughout measurement, which is thought to cause bias but has not been addressed due to the difficulty of measuring mesophyll conductance (*g_m_*) under low light intensities.

**Figure 2.**
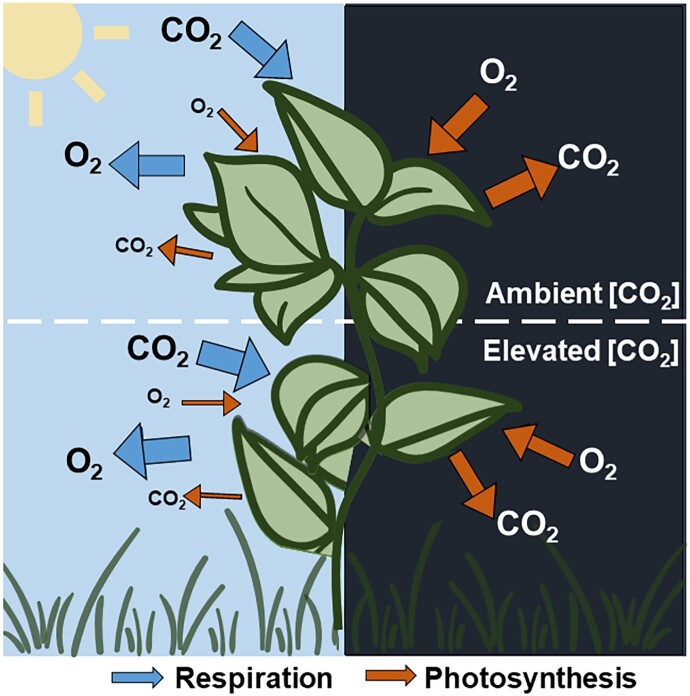
Processes affecting NPP during the day and night under ambient and elevated carbon dioxide concentrations (CO_2_). Arrow sizes indicate the relative rate of each process, with both day-time (*R_l_*) and night-time (*R_d_*) respiration decreasing under elevated (CO_2_).

In this issue of *Plant Physiology,*Sun et al. (2023) explore the short- and long-term responses of leaf day respiration to ambient (410 ppm) and elevated (820 ppm) (CO_2_) in wheat (*Triticum aestivum*) and sunflower (*Helianthus annuus*). Using the Kok method, the Kok-phi method, and a modified Kok method [termed by the authors as the Kok-Cc method that accounts for changes in chloroplastic (CO_2_)], they identified, on average, an 8.4% reduction in *R_l_* and a 16% reduction in *R_d_* undergrowth at elevated (CO_2_) (Fig. 2). However, the authors did not identify any significant change in the *R_l_*: *R_d_* ratio between each treatment. During a short-term change in (CO_2_) during measurement, Sun et al. (2023) found an increase in *R_l_* and the *R_l_*: *R_d_* ratio using the Kok and Kok-Phi method but not the Kok-Cc method, a discrepancy attributed to changes arising from differences in intercellular (CO_2_). This indicates a tendency to underestimate *R_l_* and overestimate light inhibition under low light intensities using the Kok and Kok-Phi methods. Incorporating intercellular (CO_2_) into the methodology indicates that light inhibition of respiration is approximately 6 ± 4%, equivalent to 26% of the total Kok effect.

Similar to previous results, including those from free-air CO_2_ enrichment studies (Ainsworth and Long 2005), Sun et al. (2023) found a reduction in both leaf N and chlorophyl content under elevated (CO_2_). Thus the concurrent reduction in *R_l_* and *R_d_* is linked to changes in N metabolism in leaves. Together this indicates a complex relationship between atmospheric CO_2_, C-, and N- cycles. Whilst the modified Kok-Cc method presented by Sun et al. (2023) presents a more reliable approach toward the assessment of plant respiration, theoretical difficulties still arise due to measurement under low ambient light intensities. Thus, whilst we are now one step closer to understanding plant function in future environments, further work is needed to determine the response of *R_l_* to changes in irradiance.
